# Prevalence of undiagnosed hypertension and associated factors among adults in Durame town, Southern Ethiopia: a cross-sectional study

**DOI:** 10.3389/fepid.2023.1205857

**Published:** 2023-11-27

**Authors:** Abebe Suliman, Sebsibe Tadesse, Lonsako Abute, Markos Selamu

**Affiliations:** ^1^ School of Public Health, Wachemo University, Hossana, Ethiopia; ^2^ National Data Management and Analytics Center for Health, Ethiopian Public Health Institute, Addis Ababa, Ethiopia

**Keywords:** alcohol consumption, family history of hypertension, health-seeking behavior, hypertension, physical activity, street foods, undiagnosed hypertension

## Abstract

**Background:**

Undiagnosed hypertension is a major public health problem causing severe cardiovascular disorders that are responsible for a high proportion of morbidities and mortalities, especially among adults living in low-income countries. However, there is a paucity of information that describes its epidemiology in Ethiopia. This study aimed to assess the prevalence of undiagnosed hypertension and associated factors among adults in Durame town, southern Ethiopia.

**Methods:**

A community-based cross-sectional study was conducted from July to September 2022. Data were collected from 526 randomly selected adults aged ≥18 years using a pre-tested questionnaire. The binary logistic regression models were used to identify factors associated with undiagnosed hypertension.

**Results:**

The prevalence of undiagnosed hypertension among adults in Durame town, southern Ethiopia, was found to be 14.0% (95% CI: 11.2–17.1). Family history of hypertension [AOR = 6.9, 95% CI: (3.62, 13.27)], drinking too much alcohol [AOR = 5.7, 95% CI: (2.97, 10.75)], physical inactivity [AOR = 2.5, 95% CI: (1.34, 4.73)], consuming street foods regularly [AOR = 2.8, 95% CI: (1.28, 6.01)], and seeking healthcare for hypertensive symptoms without serious illness [AOR = 2.4, 95% CI: (1.28, 4.56)] were significantly associated with developing undiagnosed hypertension.

**Conclusion:**

The study has revealed that one in seven adults had undiagnosed hypertension in the study area. Thus, interventions to prevent hypertension should target increasing awareness among people with a family history of hypertension, controlling excessive alcohol consumption, promoting physical exercise, regulating street food markets, and improving the health-seeking behavior of adults in urban settings.

## Background

Hypertension is a state of systemic blood pressure defined by a systolic blood pressure of at least 140 mmHg and/or a diastolic blood pressure of at least 90 mmHg on two or more consecutive readings (1). In 2019, there were an estimated 1.28 billion adults aged 30–79 years with hypertension globally; about two-thirds of them live in low- and middle-income countries (2). Studies have revealed that the risk of developing hypertension among people living in Subsaharan Africa is increasing, possibly due to poor socioeconomic conditions, weak health infrastructure, an increasing rate of urbanization, a sedentary lifestyle, and poor nutrition (3).

There are significant gaps in hypertension diagnosis, treatment, and control strategies in Subsaharan Africa (4). A large proportion of people with hypertension remain undiagnosed for years, causing premature deaths and serious health complications such as heart failure, renal failure, stroke, and myocardial infarction. The pooled prevalence of undiagnosed hypertension was estimated to be 30%. Of those with hypertension, about 73% were unaware of their hypertensive status, 82% were not receiving treatment, and 93% did not control their blood pressure (5).

Prevention and control of hypertension continue to be a major public health challenge in Ethiopia due to a lack of transportation, a poor referral system, misconceptions about symptoms, a lack of financial and social support, and traditional healing practices (6). According to the 2016 national survey, about 77% of the participants reported that their blood pressure was never measured (7). In 2019, the hypertension treatment rate among women was only 16%, the sixth lowest rate globally (8). Moreover, the magnitude of the risk factors for hypertension, like obesity, physical inactivity, stress, unhealthy diets, tobacco use, and alcohol consumption, is increasing, especially among urban communities (9). However, there is a paucity of studies that explain the epidemiology of undiagnosed hypertension in the country. Therefore, this study was intended to assess the prevalence of undiagnosed hypertension and associated factors among adults in Durame town, southern Ethiopia. The study will provide the latest information for policymakers, planners, and programmers to design appropriate hypertension prevention and control strategies.

## Methods

### Study design and area

This community-based cross-sectional study was conducted in Durame town, southern Ethiopia, from July to September 2022. The town had an estimated population of 52,513 living in three kebeles (the smallest administrative unit in Ethiopia). There is one general hospital, three health centers, nine clinics, one health post, and nine drug stores (10).

### Participants and data collection

All randomly selected adults aged ≥18 years who lived in the study area for at least six months without a history of hypertension and did not visit any health facility for a blood pressure check in the past year were included in the study. Adults who were unable to respond to the interview questions and/or take physical measurements were excluded from the study. Data on socio-demographic characteristics, family history of hypertension, behavioral characteristics, physical activity, dietary practices, and health-seeking behaviors were collected using a pre-tested questionnaire.

After completing the interview, the data collectors measured the participants' weight and height. The weight was measured by using a portable digital weight scale and recorded to the nearest 0.1 kg. The height was measured by using a portable Stadiometer and recorded to the nearest 0.1 cm. The participants were instructed to stand on the footplate with their backs to the Stadiometer, their legs straight, their arms at their sides, and their shoulders relaxed, and touch the Stadiometer at their heels, buttocks, upper back, and head. Then, the headpiece was lowered until it touched the crown of the head firmly, compressing the hair.

Blood pressure was measured using a standard mercury sphygmomanometer with a cuff size that covers two-thirds of the upper arm. No exercise, smoking, coffee, or tea were allowed 30 min before the measurement. The participants were instructed to sit on the chair with their legs uncrossed, feet on the floor, back supported, arm supported on the desk to keep the upper arm at the same level as the heart, and an empty bladder guaranteed. The upper arm was uncovered two inches above the elbow crease. A cuff is placed on the brachial artery in the upper arm. Three blood pressure measurements were taken 10 min apart in a sitting position. Finally, the average of the three readings was taken to determine the blood pressure status of the respondents.

### Data quality control

The questionnaire was translated into Amharic, the official language, and back to English to maintain consistency in meaning. The interview questionnaire was pilot tested on 26 adults living in Teza Agara that had characteristics nearly similar to Durame town in order to identify the interview questions and physical measurements that needed adjustments. The data collectors and supervisors received intensive training for two days on how to obtain ethical consent, conduct interviews, measure weight, height, and blood pressure, and keep records. The collected data were checked for consistency and completeness on a daily basis.

### Sample size calculation

Epi Info version 7 was used to determine the minimum sample size of 526 by taking the 28.8% expected proportion of undiagnosed hypertension (9), 5% margin of error, 95% confidence level, 1.5 design effects, and 10% non-response rate.

### Sampling procedure

Multistage sampling was used to recruit the study participants. In the first stage, four ketenes (villages in the kebeles) were selected from a pool of 10 ketenes by using the lottery method. The town administrative office provided the list of households in each selected ketene, which was used as a sampling frame. At the second stage, a total of 526 samples were proportionally allocated to each selected ketene (i.e., 100 for Arada ketene, 144 for Teza ketene, 139 for Arada Girige ketene, and 143 for Abonsa ketene). At the last stage, the households with the eighth interval in the sampling frame of each ketene were selected for the data collection. If more than one eligible adult was present in the selected household, one household member was selected randomly using the lottery method.

### Data management and statistical analyses

The data were entered into EpiData version 4.6 and analyzed on SPSS version 26. Frequency and percentage were used to present categorical data, whereas mean and standard deviation were used to present continuous data. All independent variables were fitted separately into a bivariate logistic regression model to estimate the degree of association with undiagnosed hypertension among adults. Then, variables with a *p*-value  <  0.25 were exported to a multivariable logistic regression model to control confounders. The model's fitness was checked by the Hosmer-Lemeshow goodness-of-fit test. A multicollinearity test was also done by examining the standard error (SE) for the b coefficient. A SE > 2 indicates the presence of multicollinearity among independent variables. In this case, SE was less than 2 for all variables. The statistical significance was declared by using an odds ratio (OR) with a 95% confidence interval (CI).

### Operational definitions

**Undiagnosed hypertension:** refers to a systolic blood pressure of ≥140 mmHg or a diastolic blood pressure of ≥90 mmHg and/or both (1), without a prior history of hypertension, and having not visited any health facility for a blood pressure check in the past year. The average of three sphygmomanometer readings taken 10 min apart in a sitting position was used to determine the blood pressure status.

**Prehypertension:** refers to a systolic blood pressure of 120–139 mmHg or a diastolic pressure of 80–89 mmHg and/or both (1).

**Normal blood pressure:** refers to a systolic blood pressure between 90 and 120 mmHg and a diastolic blood pressure between 60 and 80 mmHg (1).

**Body mass index:** refers to a quotient of weight divided by height squared (kg/m^2^).

**Drank too much alcohol:** refers to adults who drank over 330 ml of beer (5% alcohol), 330 to 750 ml of wine (20%–25% alcohol), or 200–250 ml of local alcohol, like tella (8%–15% alcohol), tej (10%–13% alcohol), or arake (40%–45% alcohol), per week (1).

**Family history of hypertension:** refers to a history of hypertension in the father, mother, or both as reported by the respondent.

**Low consumption of fruits and/or vegetables**: refers to adults who ate fruits and/or vegetables less than five times per day. One serving is equivalent to one apple, orange, or banana, or three tablespoons of cooked vegetables (1).

**Physical inactivity:** refers to adults who undertake less than 150 min of moderate-intensity physical activity per week, less than 75 min of vigorous-intensity physical activity per week, or a combination of both (11).

**Street foods**: refers to foods and beverages that were prepared and sold by vendors in streets and public places for consumption.

## Results

### Socio-demographic characteristics

A total of 516 adults participated in the study, making the response rate 98.1%. Of whom, 50.8% were males. The mean age with a standard deviation was 34 ± 11. Nearly half, 49.6%, of the participants belonged to the age group of 30–49 years. The majority, 82.0%, of the participants were married. About 47.3% of the participants had a high school or higher certificate. Regarding occupation, the proportion of government/self-employed and farmers was 37.8% and 33.3%, respectively. About half, 49.6%, of the participants earned a monthly income of less than 2,300 birr (Table 1).

**Table 1:** Socio-demographic characteristics of adults in Durame town, southern Ethiopia, 2022.

Variable	Frequency	Percent
Sex		
Male	262	50.8
Female	254	49.2
Age (in years)		
18–29	200	38.8
30–49	256	49.6
≥50	60	11.6
Marital status		
Married	423	82.0
Single	73	14.1
Other	20	3.9
Educational status		
Had no formal education	49	9.5
Primary school	223	43.2
High school and above	244	47.3
Occupation status		
Farmer	172	33.3
Government/self employed	195	37.8
Other	149	28.9
Monthly income (in birr)		
<2,300	256	49.6
≥2,300	260	50.4

### Behavioural characteristics

The majority, 93.4%, of the participants did not use any tobacco products. A nearly similar proportion, 94.4%, of them did not smoke cigarettes in the past 30 days. About three-fourths of the participants did not drink alcohol during the data collection. Beer was a favourite type of alcoholic drink for nearly half, 52.0%, of the participants. The majority, 81.2%, of the participants did not drink too much alcohol. The proportion of those who did not chew khat was 85.7% (Table 2).

**Table 2:** Behavioural characteristics of adults in Durame town, southern Ethiopia, 2022.

Variable	Frequency	Percent
Used any tobacco products		
No	482	93.4
Yes	34	6.6
Smoked cigarettes in the past 30 days		
No	487	94.4
Yes	29	5.6
Currently drinking		
No	385	75.2
Yes	127	24.8
Favourite type of alcohol		
Beer	66	52.0
Wine	16	12.6
Tej	29	22.8
Areke	16	12.6
Drank too much alcohol		
No	419	81.2
Yes	97	18.8
Chewed khat		
No	442	85.7
Yes	74	14.3

### Participants' knowledge about hypertension

All adults who participated in this study heard about hypertension. The main source of information about hypertension was health institutions, which accounted for 60.7%. The major cause of hypertension was reported to be stress (52.8%), followed by high salt and fat consumption (30%). More than two-thirds, or 70%, of the participants reported that hypertension could not be prevented. The majority, 60.3%, of the participants did not report that hypertension could cause serious health complications. Less than one-third, or 30.8%, of the participants reported that regular measurement of blood pressure was important to prevent hypertension (Table 3).

**Table 3:** Hypertension knowledge of adults in Durame town, southern Ethiopia, 2022.

Variable	Frequency	Percent
Main source of information about hypertension		
Health institutions	313	60.7
Friends	85	16.5
Media	44	8.5
Others	74	14.3
Causes of hypertension		
Stress	251	52.8
Consuming high salt and fat	147	31.0
Drinking alcohol	53	11.2
Smoking	24	5.0
Hypertension can be prevented		
No	361	70.0
Yes	155	30.0
Hypertension can cause serious health complication		
No	311	60.3
Yes	205	39.7
Regular measurement of blood pressure is important		
No	357	69.2
Yes	159	30.8

### Prevalence of undiagnosed hypertension

The mean systolic blood pressure with a standard deviation was 113 ± 23 mmHg, and the mean diastolic blood pressure with a standard deviation was 76 ± 10 mmHg. The distribution of blood pressure was observed to be higher than these among participants in the age group of 30–49 years, attended primary school and below, had a family history of hypertension, physically inactive, drank too much alcohol, drank tej, areke, wine and beer, chewed khat, and consumed street foods regularly (Table 4). The prevalence of undiagnosed hypertension was 14% (95% CI: 11.2–17.1%), whereas the prevalence of prehypertension was 10.1% (95% CI: 7.8–12.8%).

**Table 4:** Distribution of systolic and diastolic blood pressure of adults in Durame town, southern Ethiopia, 2022.

Variable	Systolic blood pressure	Diastolic blood pressure
	(standard deviation)	(standard deviation)
Sex		
Male	113 (21)	76 (10)
Female	112 (24)	76 (10)
Age (in years)		
18–29	110 (20)	75 (9)
30–49	115 (24)	77 (10)
≥50	113 (24)	76 (10)
Educational status		
Had no formal education	116 (27)	77 (10)
Primary school	114 (22)	77 (10)
High school and above	111 (22)	76 (10)
Family history of hypertension		
No	110 (19)	75 (9)
Yes	125 (30)	82 (12)
Physical inactivity		
No	109 (20)	75 (9)
Yes	117 (25)	78 (11)
Drank too much alcohol		
No	109 (21)	75 (9)
Yes	127 (24)	83 (11)
Favourite type of alcohol		
Beer	125 (26)	81 (10)
Wine	127 (24)	83 (11)
Tej	137 (15)	88 (10)
Areke	132 (20)	86 (9)
Chewed khat		
No	112 (22)	76 (10)
Yes	119 (26)	79 (10)
Consumed street foods regularly		
No	108 (18)	74 (9)
Yes	115 (24)	77 (11)

### Factors associated with undiagnosed hypertension

Age, occupation status, family history of hypertension, drinking too much alcohol, chewing khat, physical inactivity, low consumption of fruits and/or vegetables, body mass index, consuming street foods, and healthcare for hypertensive symptoms without serious illness showed association with undiagnosed hypertension in the bivariate analysis at *p*-value < 0.25. Family history of hypertension [Adjust OR (AOR)  =  6.9, 95% CI: (3.62, 13.27)], drinking too much alcohol [AOR = 5.7, 95% CI: (2.97, 10.75)], physical inactivity [AOR = 2.5, 95% CI: (1.34, 4.73)], consuming street foods regularly [AOR = 2.8, 95% CI: (1.28, 6.01)], and seeking healthcare for hypertensive symptoms without serious illness [AOR = 2.4, 95% CI: (1.28, 4.56)] remained significant in the multivariable logistic regression model (Table 5).

**Table 5:** Factors associated with undiagnosed hypertension in Durame town, southern Ethiopia, 2022.

Variables	Undiagnosed Hypertension		Crude OR (95% CI)	*p*-value	Adjusted OR (95% CI)	*p*-value
	Yes	No				
Age (in years)						
18–29	19	181	1		1	
30–49	44	212	0.6 (0.25, 1.40)	0.232	0.4 (0.14, 1.16)	0.092
≥50	9	51	1.2 (0.54, 2.6)	0.683	0.8 (0.30, 2.1)	0.641
Occupational status						
Farmer	17	155	1		1	
Government/self employed	27	168	1.4 (0.81, 2.57)	0.216	1.2 (0.57, 2.38)	0.686
Other	28	121	0.7 (0.36, 1.3)	0.246	0.5 (0.25, 1.17)	0.116
Family history of hypertension						
No	36	386	1		1	
Yes	36	58	6.7 (3.89, 11.40)	0.001	6.9 (3.62, 13.27)	<0.001
Chewed khat						
No	58	384	1		1	
Yes	14	60	1.5 (0.81, 2.94)	0.186	0.6 (0.29, 1.37)	0.241
Drank too much alcohol						
No	35	384	1		1	
Yes	37	60	6.8 (3.96, 11.57)	0.001	5.7 (2.97, 10.75)	<0.001
Physical inactivity						
No	26	279	1		1	
Yes	46	165	2.99 (1.78, 5.02)	0.001	2.5 (1.34, 4.73)	<0.004
Low fruit and/or vegetable						
No	59	396	1		1	
Yes	13	48	1.82 (0.93, 3.56)	0.081	0.9 (0.40, 2.18)	0.883
Consumed street foods regularly						
No	11	173	1		1	
Yes	61	271	3.5 (1.81, 6.92)	0.001	2.8 (1.28, 6.01)	<0.01
Body mass index (in kg/m^2^)						
<25	52	357	1		1	
≥25	20	87	1.6 (0.89, 2.78)	0.114	0.7 (0.35, 1.46)	0.356
Sought healthcare for hypertensive symptoms without serious illness						
Yes	37	149	1		1	
No	35	295	2.1 (1.27, 3.46)	0.004	2.4 (1.28, 4.56)	0.007

## Discussion

This study aimed to assess the prevalence of undiagnosed hypertension and associated factors among adults in Durame town, southern Ethiopia, in 2022. The study has revealed that the prevalence of undiagnosed hypertension was 14.0% (95% CI: 11.2–17.1%). Moreover, family history of hypertension, drinking too much alcohol, physical inactivity, consuming street foods regularly, and seeking healthcare for hypertensive symptoms without serious illness were found to be significantly associated with undiagnosed hypertension.

The prevalence of undiagnosed hypertension observed in this study was consistent with other studies conducted in Addis Ababa city, 13.25%; Hawasa city, 12.3%; Dano district, 14.6%; and Debre-Markos town, 12.7% (12–15). The possible explanation for the observed similarity might be that the study participants in all the studies were adults over the age of 18 years. People in similar age groups might share similar lifestyles, health-seeking behavior, and socio-economic contexts. However, the observed prevalence was lower than reports from Gunchire district, 18.8%; Bahir-Dar city, 24.8%; Wolaita-Sodo town, 28.8%; Sudan, 38.2%; and Lebanon, 42.69% (9, 16–19). The disparity might be due to the fact that different standards were used to define the cut-off point for hypertension. The previous studies used the World Health Organization pre-hypertension stage to define the hypertension cut-off point; however, this cut-off point was proposed to define hypertension among individuals with comorbidities (20). The socio-demographic and ethnic differences among the populations could also determine the risk of developing hypertension. Furthermore, the prevalence of undiagnosed hypertension identified in this study was higher than that reported from Gilgal-Gibe field research site (7.5%), India (10.1%), the USA (6.5%), and Iran (4.8%) (21–24). The Gilgel-Gibe study was conducted in rural settings and might have a protective effect against developing hypertension (25). Socio-economic, access to healthcare facilities, and lifestyle differences could explain the observed variation.

This study has revealed that there is a significant association between family history of hypertension and undiagnosed hypertension. The odds of developing hypertension among adults who had a family history of hypertension were seven times higher among those who had not. This was consistent with reports from other locations in Ethiopia, like Hawassa city, Dano district, and Debre-Markos town (12, 13, 15, 26). This could be explained by the fact that family members might share similar risk factors and inherit genetic traits for developing hypertension. Thus, community-based interventions to reduce the burden of hypertension should include creating awareness among people with a family history of hypertension.

This study has revealed that the odds of developing hypertension among those adults who drank too much alcohol were nearly six times higher compared to those who did not. This was in agreement with studies from Ethiopia and Japan (16, 27–30). Hence, disseminating health information on the harmful effects of excessive alcohol consumption, restricting alcohol advertisements, and decreasing the availability and accessibility of alcohol through increasing alcohol taxation and regulating alcohol sales can be recommended.

Insufficient physical activity is one of the major modifiable risk factors for hypertension. In this study, the odds of developing hypertension among adults who did not engage in sufficient physical activity were about three times higher than those who did. Previous studies from Bahir-Dar city, Gunchire district, and Hosanna town supported this evidence (9, 19, 31). Physical activity is not at a moderate level in urban areas of Ethiopia because of increasing urbanization, and people usually use taxes and motor cycles to move from place to place (7, 32). This suggests that urban public health programs should target promoting physical exercise among community members to reduce the risk of developing hypertension.

This study has indicated that the odds of developing hypertension among adults who consumed street foods regularly were about three times higher compared to those who did not. This was consistent with reports from Tanzania and Italy (33, 34). This could be due to the fact that street foods usually have a high quantity of carbohydrate, saturated fat, and sodium; thus, consuming these foods might contribute to the development of undiagnosed hypertension (35). This indicated that there is a need to regulate street food markets in urban settings.

The odds of developing hypertension among adults who did not seek healthcare for hypertensive symptoms without serious illness were about two times higher than those who did. This was consistent with studies from Dano and Gunchire districts, Ethiopia (9, 13). Many people living in resource-limited countries practice prolonged self-treatment as the first step in the health-seeking process. Therefore, strengthening the health extension program can improve the health-seeking behaviour of people living in resource-limited settings.

This study has limitations. Establishing a cause-and-effect relationship between the independent variables and hypertension was difficult because of the cross-sectional nature of the study. The other limitation was that the study measured blood pressure three times in one visit, which differed from the clinical practice guidelines recommendation of two visits (1, 36). Moreover, the blood pressure measurement was limited to only behavioural and physical measurements of the participants and did not include biochemical measurements.

## Conclusion

The study has revealed that one in seven adults had undiagnosed hypertension in the study area. Thus, interventions to prevent hypertension should target increasing awareness of people with family histories of hypertension, controlling excessive alcohol consumption, promoting physical exercise, regulating street food markets, and improving the health-seeking behavior of adults in urban settings.

## Data Availability

The original contributions presented in the study are included in the article/Supplementary Material, further inquiries can be directed to the corresponding author.
